# A feedback loop between management, intraspecific trait variation and harvesting practices

**DOI:** 10.1093/aobpla/plad077

**Published:** 2023-11-16

**Authors:** Jonathan Locqueville, Cyrille Violle, Doyle McKey, Sophie Caillon, Sylvain Coq

**Affiliations:** Centre d'Ecologie Fonctionnelle et Evolutive, Univ Montpellier, CNRS EPHE, IRD, Montpellier 34293, France; Centre d'Ecologie Fonctionnelle et Evolutive, Univ Montpellier, CNRS EPHE, IRD, Montpellier 34293, France; Centre d'Ecologie Fonctionnelle et Evolutive, Univ Montpellier, CNRS EPHE, IRD, Montpellier 34293, France; Centre d'Ecologie Fonctionnelle et Evolutive, Univ Montpellier, CNRS EPHE, IRD, Montpellier 34293, France; Centre d'Ecologie Fonctionnelle et Evolutive, Univ Montpellier, CNRS EPHE, IRD, Montpellier 34293, France

**Keywords:** Grassland, grazing, habitat management, intraspecific trait variation, medicinal and aromatic plants, mowing, rangeland management

## Abstract

Abstract. Intraspecific variation in plants is a major ecological mechanism whose local determinants are still poorly understood. In particular, the relationship between this variation and human practices may be key to understanding human–nature relationships. We argue that it is necessary to consider how human practices both influence and depend on the phenotypic variability of species of interest. *Arnica montana* (arnica) is a good model to study the complex interactions between human actions and plant phenotype, as (i) its ecological niche is shaped by human management actions and (ii) its variability has consequences for harvesters. Using a functional trait approach, we examined feedback loops linking management actions, plant phenotype and harvesting practices. In 27 sites in southeastern France, we measured vegetative and reproductive functional traits of arnica of interest for harvesters, and recorded management actions (grazing; mowing) and ecological variables (including height of surrounding vegetation and tree cover). We examined their effects on plant traits with linear mixed models and used path analysis to test if the effects of human management on traits are mediated by the height of surrounding vegetation. Management actions affected functional traits of arnica. Biomass removal practices (grazing, mowing) were associated with smaller plants producing smaller leaves with reduced specific leaf area. We uncovered the core role of the height of surrounding vegetation in determining this phenotype. Tree cover was associated with reduced flowering. The observed intraspecific variation in response to management actions differentially impacts the two main harvesting practices. Flower-head harvesting depends on reproductive traits that are not impacted by mowing (which is done in winter) but adversely affected by tree cover. In contrast, traits associated with large biomass under tree cover or with high surrounding vegetation are favourable for whole-plant harvesters. Our trait-based approach unveiled clear links between management actions and plant phenotype, with impacts on both vegetative and reproductive traits. These changes induced by management also affect the practices of harvesters. We thus demonstrated a feedback loop between human actions and plant phenotype and provided a novel perspective on human-related causes and consequences of plant intraspecific variability.

## Teaser

In this paper, we demonstrate the existence of a feedback loop involving rangeland management practices, *Arnica montana* phenotype and harvesting practices. We show that vegetative and reproductive traits are differentially affected by management, thus impacting whole-plant harvesting differently than flower-head harvesting alone. This highlights the importance of human-related causes and consequences of intraspecific variability in plants.

## Introduction

Intraspecific trait variation (ITV), that is, phenotypic differences between individuals of the same species, is, along with the turnover of species, a major mechanism underpinning the link between environmental conditions and plant functional traits (Garnier *et al.* 2016). In recent years, many studies have explored both causes and consequences of ITV at several scales. Ecological causes of ITV have largely been explored through quantifying the magnitude of this variation at different scales (Albert *et al.* 2011; Violle *et al.* 2012; Siefert *et al.* 2015), but large knowledge gaps remain on the determinants of this variation at the local scale. Ecological effects of ITV have been evaluated with respect to several patterns and processes such as coexistence in plant community assemblages (Jung *et al.* 2010), community structure and primary productivity (Raffard *et al.* 2019), prey community structure (Tielens and Gruner 2020), litter decomposition (Coq *et al.* 2018) and nutrient cycling (Lecerf and Chauvet 2008).

**Figure 1.**
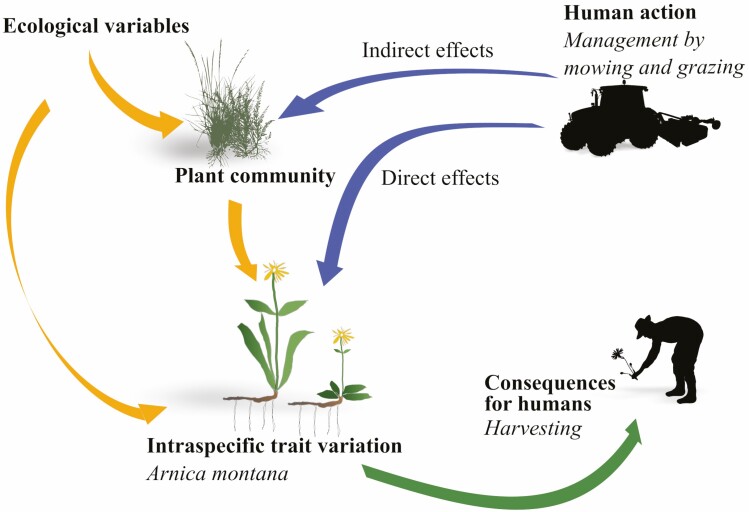
Conceptual framework of the feedback loop involving the intraspecific variation taken both as an ecological response to human disturbance and as an effect on human practices such as harvesting. The specific management actions and consequences for humans to which we apply this concept are shown in italics. Three arrows on the left: effects of ecological variables on *A. montana* and the plant community; two arrows on the top right: effects of human management; arrow on the lower right: contribution of *A. montana* intraspecific variation to human activities.

In recent years, ecology has been marked by an increased attention to ecosystems shaped by human actions. Effectively integrating the understanding of human activities and ecological processes is, therefore, a major challenge (Guerrero *et al.* 2018). In the case of intraspecific variability, many gaps remain when it comes to the integration of human practices. On the one hand (Fig. 1: blue arrows), human practices, by influencing ecological processes, impact the intraspecific diversity of plants. For example, variation of soil compaction generated ITV in the leaf resource conservation strategy of grapevine (Martin *et al.* 2022); tillage practices affected the spatial distribution of maize roots (Wang *et al.* 2015); increased intensity of grassland land use increased root P and N at the intraspecific level (Herz *et al.* 2017) and short-term management changes in meadows caused changes in height and biomass partly explained by ITV (Volf *et al.* 2016). How this variation is affected by microenvironmental heterogeneity, species interactions and disturbances is still poorly understood (Shipley *et al.* 2016), making it difficult to develop functional models of plant communities under human disturbance regimes. On the other hand (Fig. 1: green arrow), intraspecific variation, which includes genetic and phenotypic diversity among individuals of a given species, provides important contributions to humans, notably in terms of ecosystem resilience, food security and medicine (Des Roches *et al.* 2021). ITV in cultivated plants can increase yield security (e.g. Creissen *et al.* 2016) and nutritional diversity (de Haan 2019) and can allow humans to take advantage of different ecological compartments of the landscape (Locqueville *et al.* 2022). Evidence of human benefits from ITV is scarcer in natural settings and includes mainly indirect effects through the provision of ecosystem services. For example, higher ITV in *Zostera marina* was associated with higher nutrient retention (Reynolds *et al.* 2012). In another example, rate of leaf decomposition by an aquatic fungus increased with ITV (Duarte *et al.* 2019). ITV in chemical traits (e.g. phenolic compounds varying between chemotypes in *Thymus vulgaris*; Thompson *et al.* 2003) also provides a critical pharmacological resource. We argue that when studying plant ecology in a context of human–plant interdependence, it is necessary to consider how human practices both influence and depend on the phenotypic variability of the species of interest (Fig. 1).

*Arnica montana* (Asteraceae) provides a good model to study human-related causes and consequences of plant intraspecific variability. First, this plant is associated with environments created by extensive pastoralism (Kahmen and Poschlod 2000), and its ecological niche is, therefore, largely shaped by human management actions. In agropastoral systems where it thrives, the main management practices that affect vegetation traits are fertilization and the regime of disturbance of the canopy by removal of aboveground vegetation (including arnica itself), such as through grazing or mowing (Garnier and Navas 2012) and in some cases burning (te Beest 2021). In particular, the regime of canopy removal is likely to affect vegetation height and to favour plants with traits leading to higher growth rates (strategy of tolerance to herbivory), or on the contrary selecting plants with traits leading to lower palatability (McIntyre *et al.* 1999; Díaz *et al.* 2001; da Silveira Pontes 2015), depending in particular on soil fertility (da Silveira Pontes 2015). Previous studies on population ecology and conservation of arnica demonstrated, in particular, the strong sensitivity of this plant, adapted to oligotrophic habitats, to nutrient enrichment (Hollmann *et al.* 2020), and to the vegetation changes that accompany land abandonment (Vikane *et al.* 2019). To what extent intraspecific trait variability in arnica is associated with these management regimes is not well understood, but some studies have highlighted its high phenotypic variability (Romero *et al.* 2011; Vera 2014), which may be linked to environmental gradients and management regimes. Management-induced plant variability is also expected to have substantial consequences for human societies, as arnica is also a major medicinal plant of the European pharmacopoeia, and harvesting in the wild is today the main mode of supply. Intraspecific variation in the phenotype of arnica, in particular in response to rangeland management, is central for harvesting practices, because arnica is harvested under two different modalities. Harvesters pick either the flower head alone or the whole flowering plant. The flower head is used for oily macerations and in the cosmetic industry, while the whole plant is used in alcoholic extraction for the pharmaceutical and homeopathic industries. The latter form involves picking the flowering stem with its basal rosette and a small piece of rhizome and roots. Therefore, these two harvesting methods interact differently with vegetative and reproductive traits of the plant. Picking the flower heads is feasible only when plants flower densely, with a high allocation to reproduction, while picking the whole flowering plant requires a high biomass of the flowering rosettes. Harvesters’ activity is thus impacted by ITV in both vegetative and reproductive traits. Reproductive traits, however, are frequently overlooked in the study of vegetation response to management. While a number of studies have shown positive allometric relationships between plant size and reproductive output (Weiner *et al.* 2009), the relationship between particular vegetative and reproductive traits has rarely been studied.

In this study, we aimed at unveiling the local determinants of intraspecific variability in arnica, in particular, in response to management regimes, and the implications of this ITV for harvesters. Specifically, in the Monts d’Ardèche region, France, we performed a comprehensive study linking management actions and the phenotypic response of arnica to them, with a particular focus on traits of potential importance for harvesters. We analysed the response of both reproductive and vegetative traits to tree cover and to two regimes of canopy removal, grazing and mowing and how these traits vary in relation to each other. Since both flower heads and vegetative parts are picked, we investigated several candidate traits: traits related to plant biomass (leaf fresh mass, reproductive height) and traits important for flower picking (percentage of rosettes flowering and number of flowers per rosette). We also investigated other functional traits of ecological importance, which are detailed below.

## Material and Methods

### Focal species

*Arnica montana* is a clonal perennial herbaceous plant, typical of oligotrophic to oligo-mesotrophic, acidophilic to neutrocline heaths and meadows (Luijten *et al.* 1996), and margins and openings of forests (Sugier *et al.* 2019). In France, the main populations are located between 1200 and 2500 masl. The amount collected annually is unknown, and past estimations (Lange 1998: 50 metric tons of dry flower heads in Europe) did not include whole-plant harvesting, which today accounts for a large proportion of the production. Today this amount is supplied partly by wild collection and partly by cultivation. This species has been listed in the Annex V of the European Union (EU) Habitats Directive 92/43/EEC as a species of community interest, whose exploitation may be managed and whose conservation should be encouraged.

### Study site and sampling

We selected 27 sites in the Monts d’Ardèche region of France, located between 44.62 and 44.94 °N; 3.97 and 4.28 °E (Supporting Information—Fig. S1), with site elevation ranging from 1161 to 1604 masl. Sites were selected based on the knowledge of local gatherers and our own experience in order to cover a maximum diversity of sites in terms of vegetation characteristics. Habitats included nutrient-poor grasslands and heathlands and wet meadows, as well as clearings in edges of *Pinus sylvestris* and *Fagus sylvatica* forests. All selected sites had to contain arnica, but its density was not a criterion. Permission to conduct the ecological surveys was obtained from all owners and/or managers (none were harvesters). All ecological surveys described below were carried out between 28 June and 18 July 2021, during the flowering season of arnica and before any management practice or harvesting.

To understand the effects of management actions on arnica traits, we asked the owner or manager if the site had been grazed or mowed (with a rotary mower) in the past 2 years. We recorded the answer as a yes/no variable. Among the 27 sites, 15 had a biomass removal treatment (5 had been mowed, 8 had been grazed, and 2 had been both mowed and grazed) and 12 were unmanaged (no biomass removal). The two sites that were both mowed and grazed were treated in the analyses as belonging to both categories. Only summer extensive grazing from mid-July to mid-September (after arnica flowering) was performed on the selected sites. Mowing is done in winter and is thus not expected to damage rosettes of arnica plants, which do not persist during winter.

At each site, a vegetation survey was conducted on five 1 m² quadrats containing arnica (13 ± 9 % of the total cover). The same quadrats were used for all the other surveys. Percent cover of each plant species was estimated visually and their layer was recorded. Plants having respectively a height of ≥ 2 m, 1–2 m and < 1 m were classified, respectively, in the tree, shrub and herb layers. The ‘herb’ layer could contain both herbaceous and woody plants. Accuracy of the estimation was controlled by comparing the surveys made by two observers. Total cover from the different layers was allowed to sum up to more than 100 %. Shannon indices were computed on relative covers of plants from the herb layer only. Soil depth from the surface to bedrock was measured with an auger and three replicate values per site were averaged. The nature of the bedrock (granitic or volcanic) was recorded. To distinguish sites based on the influence of tree cover, we assigned the class ‘presence of tree cover’ to sites presenting tree cover > 15 %, concerning *n* = 6 sites (four unmanaged sites and two grazed sites). The location of the quadrats was chosen randomly. The quadrat frame was moved in a random direction from the preceding quadrat to a distance of at least 6 m until the new location contained arnica.

From each quadrat, a biomass sample was collected from a 0.25 m × 0.25 m square taken outside any arnica rosette clusters, then translated to a value of standing biomass in dry weight per m², and the average height of the vegetation was estimated following the protocol recommended by Perez-Harguindeguy *et al.* (2016), and averaging three measurements in each quadrat. Counts of reproductive and vegetative variables were done at the quadrat level: the number of arnica rosettes, the number of flowering stems and the number of flower heads in each quadrat were recorded. Then, measurements were taken at the rosette level, on two randomly selected flowering rosettes in the quadrat. If no flowering rosette was present, vegetative measurements were made on a non-flowering rosette. As arnica reproduces vegetatively with a phalanx strategy (*sensu*Grime 1979), with a lateral spread of 0.01–0.25 m.yr^−1^ (Kutschera and Lichtenegger 1992), it is relatively likely that rosettes close to one another correspond to ramets of the same genet (genetic individual). Therefore, we chose the pair of rosettes closest to points A and B located at ¼ and ¾ of the diagonal line of the quadrat, so that the two rosettes were at least 50 cm apart in the quadrat. The following measurements were made on the flowering rosettes: vegetative height, reproductive height and length of first cauline leaf. Two young but fully developed basal leaves were then taken from the non-flowering rosettes directly adjacent to the selected rosette, for measurement of leaf traits.

All leaf traits were considered as describing the strategy of the plant. Among them, a subset was chosen for their putative importance in harvesting practices. The collected leaves were stored according to the protocol described by Perez-Harguindeguy *et al.* (2016) in plastic containers with the base of each leaf soaking in water and placed in the refrigerator to attain water saturation, for 24–36 h. They were then wiped dry, weighed, scanned with a flatbed scanner at 600 dpi and then dried at 60 °C for 48 h and weighed. The images obtained with the scanner were automatically processed with ImageJ to calculate the surface area of each leaf. From these data, we calculated, for each leaf, specific leaf area—SLA—(ratio between leaf area (LA)/dry weight) and leaf dry matter content (LDMC) expressed as the ratio between dry mass and fresh mass of the leaf. Carbon and nitrogen concentrations in the leaves were measured with a flash CHN Elemental Analyser (Flash EA 1112 Series; ThermoFinnigan, Milan, Italy), on dry leaves ground with a ball grinder.

### Data analysis

All analyses were performed using R v.4.0.4 (R Core Team 2020). We assigned a Raunkiaer functional type to each species through the BASEFLOR database (Julve 2021) and defined three larger functional classes: monocotyledons, woody dicotyledons and non-woody dicotyledons.

We computed average values of *A. montana* functional traits at the quadrat level. Leaf traits were first averaged to the individual level and then to the quadrat level. We performed a principal component analysis (PCA) of SLA, LDMC, LA, length of first cauline leaf (*L*_leaf1_), vegetative height (defined here as the maximum height of the rosette leaves), reproductive height, percentage of rosettes flowering, number of flower heads per rosette (number of heads divided by number of rosettes in a quadrat), leaf nitrogen content (LNC) and leaf carbon content (LCC). All bivariate relationships were tested and the *P* values were corrected for multiple test comparisons. Grime’s CSR values of arnica individuals (Grime 1977), which aim to provide an understanding of arnica strategy, and are based on the values of SLA, LA and LDMC, were computed using the globally calibrated CSR analysis tool ‘StrateFy’ (Pierce *et al.* 2017).

To estimate the effect of environmental factors and human management actions (mowing and grazing) on functional traits, we performed a linear mixed-effects model for each response trait with the R package ‘“lme4”’ (Bates *et al.* 2022). We entered environmental variables as fixed effects, and the vegetative and reproductive functional traits of *A. montana* as the response variable of the model. Bedrock type had no significant effect on any trait and was thus excluded from the analysis. We included site as a random factor to control for the hierarchical nature of our survey. To estimate the significance of each effect, a likelihood ratio test determined whether the full model was significantly better than the model without this effect (function anova in package stats; R Core Team 2020). The *P* values were adjusted for multiple comparisons using Holm step-down Bonferroni correction. Model assumptions were tested with the package DHARMa (Hartig 2022). For reproductive traits, we used a logistic regression to estimate the probability of a total absence of flowering in a quadrat; among the quadrats with flowers, we fit a generalized linear mixed model of the percentage of rosettes flowering and mean number of heads per rosette from the package glmmTMB (Brooks *et al.* 2017) with a gamma distribution and a log link.

We wished to test whether management actions affected arnica traits directly, or indirectly through their effect on the height of surrounding vegetation. Thus, we performed a path analysis (i.e. a structural equation model with only observed variables), only across sites without tree cover to avoid a confusion between tree shade and competition within the herb layer, using a diagonally weighted least squares method. Path analysis was performed with the cfa function of the ‘lavaan’ package (Rosseel 2012). Environmental variables were considered to be independent. To test the indirect (via the height of surrounding vegetation) versus direct effects, the model included, on the one hand, the effects of both height of surrounding vegetation and of the environmental variables on the trait of interest, and on the other hand the effect of only the environmental variables on the trait of interest. We tested the goodness of fit of the models by using the following indices: significance of the *χ*^2^, root mean square error of approximation (RMSEA) test, standardized root mean square residual (SRMR) and comparative fit index (CFI). Non-significant *χ*^2^ and RMSEA tests, SRMR values below 0.08 and CFI values above 0.90, indicate a good fit of the model to the data (Kline 2011).

## Results

### Environments and arnica main strategies

The percentage cover of woody dicotyledons (thus mainly chamaephytes) in the herb layer varied from 0 % to 79 %, that of monocotyledons (mainly Poaceae and Cyperaceae) varied from 0 % to 75 % and that of non-woody dicotyledons from 10 % to 88 % (Supporting Information—Fig. S2). The percentage cover of the tree layer ranged from 0 % to 100 %, the latter value corresponding to forest edges. Sampled sites contained on average 30.9 ± 8.6 plant species, and had a Shannon diversity of 2.47 ± 0.433, with a Shannon equitability index of 0.084 ± 0.018, owing to the abundance of heather (*Calluna vulgaris*, Ericaceae). Mean trait values (Table 1) indicate that the sampled arnica individuals were characterized predominantly by a C-R strategy (details in Supporting Information—Fig. S3). Coefficient of variation among quadrats was highest for reproductive traits (number of heads per rosette: 1.2; percentage of rosettes flowering: 1.0), and lowest for LCC (0.02). Among leaf traits, variation was highest for LA (0.47).

**Table 1.** Mean, standard deviation and coefficient of variation (CV) of the traits measured, at the quadrat level. Variables of interest for the harvesting of the whole plant: dark and light grey; variables of interest for the harvesting of flower heads alone: light grey. In white, other traits measured for assessing ecological strategy.

Variable	Mean	Standard deviation	CV	trait categories for harvesters.	
Leaf fresh mass (mg)	1100	410	0.37	Contribution to harvested biomass	Traits of interest for harvesters
Vegetative height (cm)	14	5.4	0.39		
Reproductive height (cm)	41	9.4	0.23		
Flower heads per rosette	0.35	0.41	1.2	Proxy of allocation to flowering	
Percentage of rosettes flowering (%)	16	16	1		
LA (leaf area; mm^2^)	3400	1400	0.41		Other traits measured for assessing ecological strategy
LDMC (leaf dry matter content; mg·g^−1^)	150	23	0.15		
SLA (specific leaf area; mm^2^·mg^−1^)	22	6.3	0.29		
*L*_leaf1_ (length of the first cauline leaf; cm)	8.6	4.2	0.49		
LNC (leaf nitrogen content; mg·g^−1^)	2	0.37	0.18		
LCC (leaf carbon content; mg·g^−1^)	41	0.83	0.02		
C score (%)	58	5.5	0.095		
S score (%)	2.9	5.6	1.9		
R score (%)	39	7.7	0.2		

### Patterns of trait covariation

**Figure 2.**
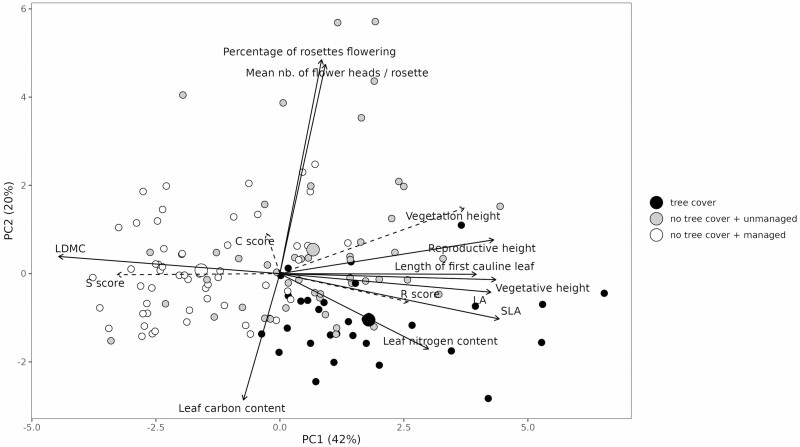
First two components of the PCA of arnica functional traits at the quadrat level. In black: with tree cover; grey: without tree cover and unmanaged (no grazing or mowing); white: without tree cover and managed (grazed or mowed). The centroid of the group is given as a wider point. Height of surrounding vegetation, Grime C, S and R scores are shown as supplementary variables.

The first three dimensions of the PCA (Fig. 2), respectively, accounted for 42 %, 20 %, and 12 % of total inertia. Leaf traits (LA, SLA, LDMC, L_leaf1_) strongly contributed to the first principal component. LA, SLA and vegetative height were strongly and positively correlated (*r* > 0.5 and *P* < 0.0001 for all pairs), and each of these was negatively correlated with LMDC (*r* < −0.5, *P* < 0.0001). In contrast, the reproductive traits were orthogonal to these leaf traits, and strongly contributed to the second component, showing a decorrelation between reproductive and vegetative traits (between mean number of heads per rosette or the percentage of rosettes flowering and LA, SLA, LDMC, vegetative height or *L*_leaf1_, *r*² < 0.04 and *P* > 0.05). Leaf nitrogen and carbon contents strongly contributed to the third component (Supporting Information—Fig. S4). Mean, standard deviation and coefficient of variation are given for all traits in Table 1.

### Determinants of the traits of interest for harvesters

#### Environmental factors

**Figure 3.**
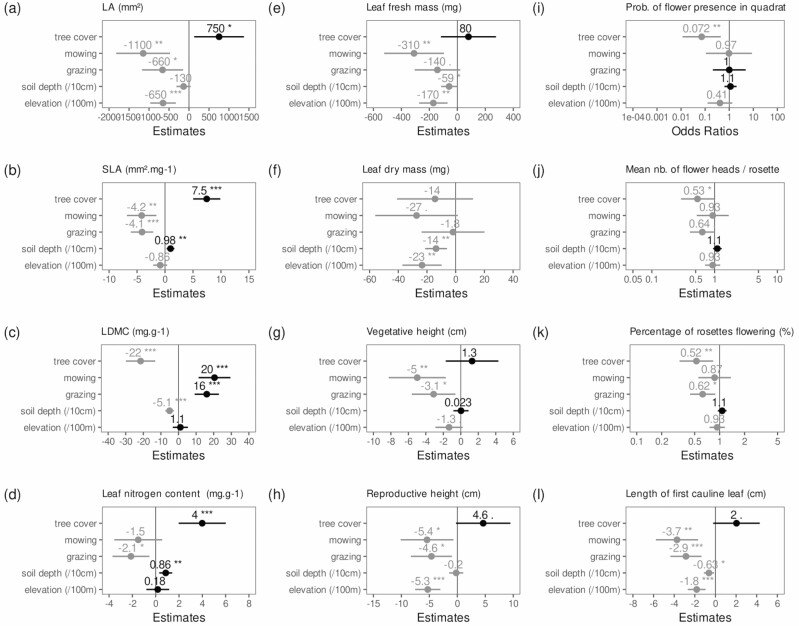
Plot of model estimates and CI for the variables of interest. Positive estimates are in black and negative estimates in grey. For binary explanatory variables (tree cover, mowing and grazing), the vertical line is the absence of the mentioned factor. Soil depth estimates are given per 10-cm unit and elevation per 100-masl unit.

Tree cover affected both reproductive and vegetative traits (Fig. 3 and Table S1): high tree cover was associated with a significantly lower percentage of rosettes flowering (*P* = 0.05) and the odds of having flowers decreased in the presence of tree cover (*P* = 0.02). Tree cover was also associated with increased LA (*P* = 0.05), SLA (*P* < 0.001) and LNC (*P* = 0.003), and decreased LDMC (*P* < 0.001). However, tree cover did not impact leaf fresh or dry mass (*P* = 0.4 and 0.6, respectively), suggesting that the positive effect on SLA is mainly due to a broader leaf and lower LDMC. Abiotic factors also had impacts on arnica functional traits. Increasing elevation had significant negative effects on several traits. Values decreased for LA (*P* = 0.002), leaf fresh mass (*P* = 0.01), leaf dry mass (*P* = 0.008) and reproductive height (*P* < 0.001). Soil depth significantly affected trait values. For each 10-cm increase in soil depth, values increased for SLA (*P* = 0.02) and LNC (*P* = 0.02), and decreased for LDMC (*P* < 0.001).

#### Management actions

Grazing and mowing had strong effects on vegetative traits (Fig. 3 and Table S1). Grazing and mowing both had strong negative effects on LA (respectively *P* = 0.05 and *P* = 0.008) and SLA (respectively *P* = 0.001 and *P* = 0.01), and positive impacts on LDMC (*P* < 0.001 for both management actions). Mowing also had significant negative impacts on vegetative height (*P* = 0.03) and leaf fresh mass (*P* = 0.03). Management actions had little effect on reproductive traits. Grazing and mowing both had marginally significant negative effects on reproductive height (*P* = 0.09 and *P* = 0.07). Grazing significantly reduced the percentage of rosettes flowering (*P* = 0.05), while mowing had no significant impact on the percentage of rosettes flowering, mean number of heads per rosette or the odds of showing no flowering.

#### Community-level effects on arnica traits

**Figure 4.**
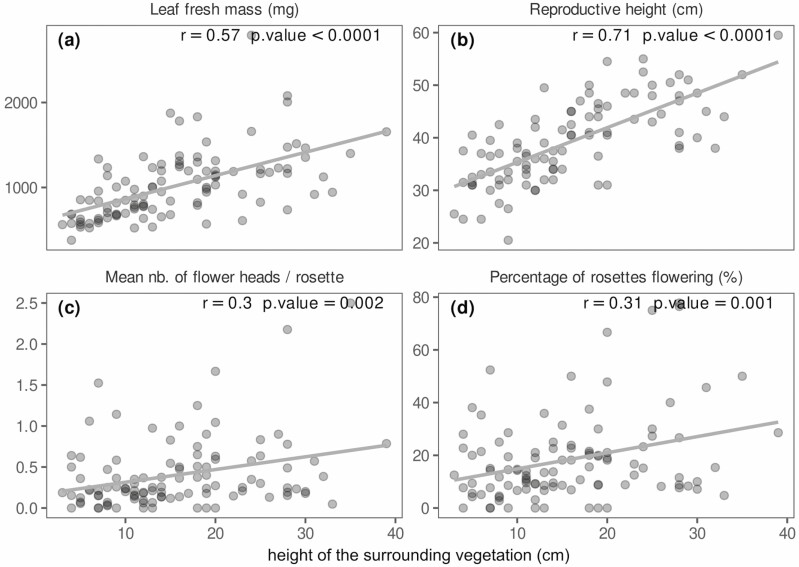
Correlations between arnica traits of interest for harvesters (leaf fresh mass, reproductive height, number of heads per rosette, percentage of rosettes flowering) and height of surrounding vegetation.

We wished to disentangle the direct effects of management actions from indirect effects mediated by altered interactions with the plant community. As arnica plants in sites with tree cover were expected to respond differently to competition, the following results include only sites without tree cover. We also focussed only on traits of interest for harvesters. In open environments, leaf fresh mass and reproductive height linearly increased with increasing height of surrounding vegetation (Fig. 4: respectively, *r* = 0.57, *P* < 0.0001; *r* = 0.71, *P* < 0.0001), suggesting that the surrounding community is one of the main factors acting on arnica traits. Correlation with reproductive traits (mean number of heads per rosette and percentage of rosettes flowering) was weaker (Fig. 4: in open environments, respectively *r* = 0.3, *P* = 0.002; and *r* = 0.31, *P* = 0.001) but suggested a positive effect of the height of surrounding vegetation on arnica allocation to flowering. Standing biomass showed a weaker correlation with arnica traits and analysis of its effects was thus included as supporting information (Supporting Information—Fig. S5).

**Figure 5.**
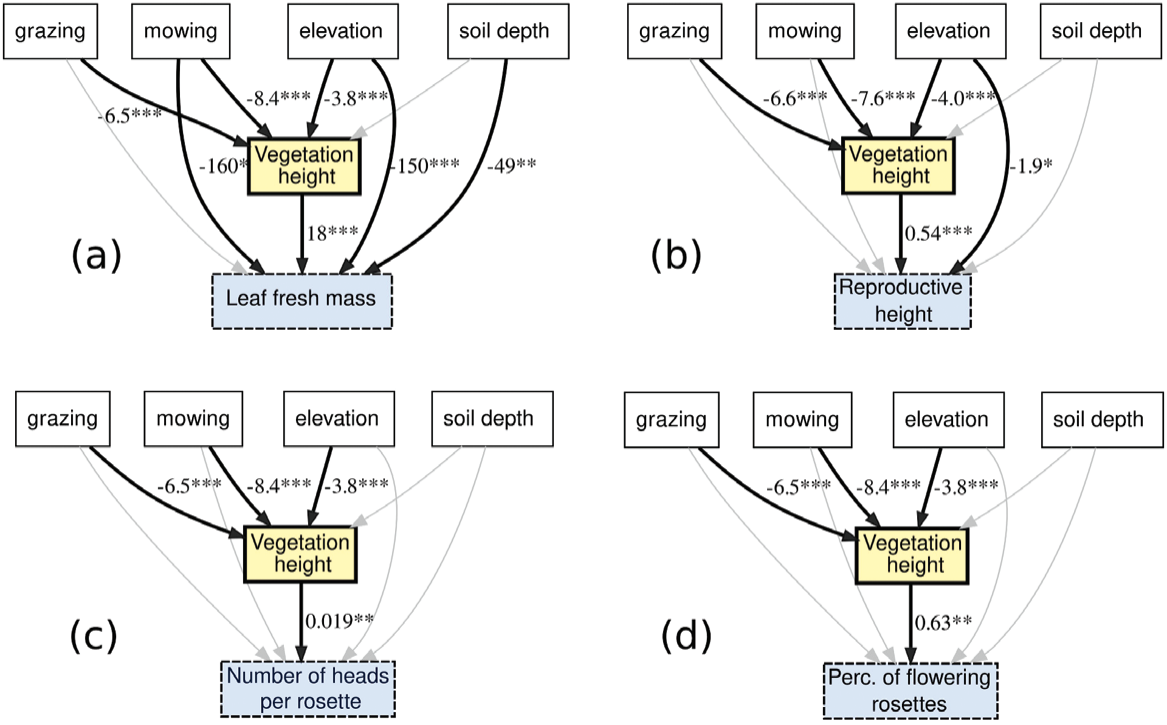
Path analysis in open environments including the effects of management actions on arnica traits of interest (leaf fresh mass, reproductive height, number of heads per rosette, percentage of rosettes flowering) and on height of surrounding vegetation. The significant effects are shown in bold lines. Estimates of the effects are expressed in the variable units and elevation estimates are given per 100-m intervals. This analysis was performed only on sites without tree cover.

The strong correlation of arnica vegetative traits with the height of surrounding vegetation suggests that the effect of management actions on these traits could be mediated by surrounding vegetation, a possibility we tested with path analysis. The model fit for the path analysis was acceptable for three of the four variables considered: leaf fresh mass, number of heads per rosette and percentage of rosettes flowering (df = 6, p(Chi2) = 0.07, CFI > 0.92, p(RMSEA) = 0.15, SRMR = 0.08 for all three variables) while for reproductive height the fit was less good (df = 6, p(Chi2) = 0.01, CFI = 0.92, p(RMSEA) = 0.04, SRMR = 0.10). The effect of mowing on leaf fresh mass was partially mediated by the height of surrounding vegetation (Fig. 5A;—8.4 × 18 = −150 mg through the mediation of vegetation height, and −160 mg as a direct effect), and the effect of grazing was fully mediated by vegetation height (−6.5 × 18 = −120 mg), as were the effects of both practices on reproductive height (mowing: −7.6 × 0.54 = −4.1 cm; grazing: −6.6 × 0.54 = −3.6 cm; Fig. 5B), on the number of heads per rosette (mowing: −8.4 × 0.019 = −0.16; grazing: −6.5 × 0.019 = −0.12; Fig. 5C) and on the percentage of flowering rosettes (mowing: −8.4 × 0.63 = −5.3 %; grazing: −6.5 × 0.63 = −4.1 %; Fig. 5D). The effect of elevation on leaf fresh mass was partially mediated by vegetation height (−3.8 × 18 = 60 mg per 100 masl increase through the mediation of vegetation height, and −150 mg as a direct effect), as was the effect of elevation on reproductive height (−2.2 cm per 100 masl increase through the mediation of vegetation height, and −1.9 cm as a direct effect). The effect of elevation on the number of heads per rosette was fully mediated by vegetation height (−0.07 heads per rosette) as was the effect of elevation on the percentage of flowering rosettes (−2.4 %). Soil depth was not found to have a significant influence on vegetation height.

## Discussion

We demonstrated that management practices strongly impacted both vegetative and reproductive traits of arnica, with feedbacks on harvesting practices. Extensive grazing and periodic re-opening of the vegetative cover by mowing are increasingly favoured by the harvesters and the organizations (in France, essentially regional parks) involved in *A. montana* conservation, to prevent canopy closure by chamaephytes and conserve arnica-rich environments. The positive impact of such practices on the demography of the plant is empirically well-known from observations by field managers. However, beyond demographic aspects, these practices are also likely to generate intraspecific variation in the phenotype of the plant, impacting the harvesting activity. Here, we demonstrated that rangeland management strongly affects a variety of plant traits, including traits that affect viability of different harvesting strategies, and these traits thus feed back to affect harvesters’ practices. While Stanik *et al.* (2020) found a low effect of management actions by mowing andgrazing on arnica traits, in our study we found that management actions in agricultural areas without trees had a remarkable effect on vegetative traits of arnica, leading to decreases in vegetative height, LA and SLA, and to an increase in LDMC. Mowing, but not grazing, was additionally found to have a negative effect on leaf fresh mass. In abandoned areas or forest edge areas, characterized by the presence of trees, tree cover also strongly impacted all leaf traits. For example, tree shade was associated with higher SLA. Increased SLA is considered to be an adaptive response to light depletion due to competition (Björkman 1981; Violle *et al.* 2009; Bennett *et al.* 2016). This is consistent with increased competition for light owing to greater light interception in forested areas than in open areas. It is also consistent with the lower SLA observed in grazed and mowed areas due to vegetation removal. In the latter case, path analyses also corroborated this explanation: the effects of management actions were partly mediated by the lower height of the vegetation in mowed and grazed areas, and were, therefore, associated with greater light availability. Interestingly, Bennett *et al.* (2016) found that LA decreased with increasing intra- and interspecific competition in controlled experiments, while we observed reduced LA when competitors were removed by grazing and mowing. One possible explanation for this difference is that the trend described by Bennett *et al.* (2016) would be a mechanism to cope with competition, while the trend we observe would be a mechanism to outcompete the other plants. Increased LA could allow the rosette leaves to cope with other species and possibly in some cases to outgrow and shade them. Altogether, our results suggest that the leaf strategy of arnica is articulated between two poles: in low-vegetation environments, its rosette plant behaviour with small leaves appressed to the ground and small cauline leaves, is characteristic of a strategy to avoid herbivory (Díaz *et al.* 2007) and canopy removal, in general. When the environment is more competitive, the winning strategy consists of increasing SLA, producing more erect rosette leaves and greatly increasing the length and surface of cauline leaves. However, it cannot be ruled out that the observed response of arnica vegetative traits to management may be generated by the effect of the canopy on unmeasured variables such as soil temperature and nutrient availability.

An original contribution of our study was to jointly examine reproductive and vegetative traits, which is rarely done in trait ecology. Interestingly, reproductive traits were found to be weakly affected by management actions, in contrast to vegetative traits. This is interesting, as it does not follow the classical pattern in which, with increasing interspecific competition, long-lived plants shift from regeneration by seeding to persistence by longevity and/or vegetative propagation (García and Zamora 2003). A possible explanation for the pattern we observed is that the level of competition was in our case not high enough to trigger a response in reproductive traits. Reproductive traits were, however, strongly affected by tree cover, with a decrease in the percentage of rosettes flowering and in the probability of flower presence. Although not affected in a statistically significant way by management actions, reproductive traits were found to be more variable overall than vegetative traits. This finding may be explained by the fact that, as a perennial plant, the investment of arnica in reproduction is less constrained, as investment can be carried over from year to year. However, comparison with other species is difficult owing to the scarcity of data reporting intraspecific variation in both vegetative and reproductive traits. Multivariate analyses of reproductive and vegetative traits of arnica showed that these two categories of traits were largely uncorrelated. This observed decoupling between reproductive and vegetative traits (Fig. 2) is noteworthy. While several studies have shown that there is a correlation between plant size and certain reproductive traits (Lechowicz and Blais 1988), there is very little information on the correlation between foliar and reproductive traits, especially at the intraspecific level. Lavorel *et al.* (1997) suggested that these two sets of traits should be analysed independently, because of the decorrelations between them. The absence of correlation despite the large amplitude observed in the leaf traits suggests that sexual reproduction of *A. montana* may be more sensitive to other factors (e.g. soil- or climate-related) than those affecting vegetative traits (i.e. more competition-related). It is useful to consider this behaviour of *A. montana* as an adaptation to the vegetation dynamics of the heathland environments where this species often thrives. The dynamics of these environments are characterized by a progressive growth of chamaephytes (*Calluna vulgaris*, *Vaccinium* spp. [both Ericaceae], *Genista* spp. [Fabaceae]) that progressively outcompete most of the herbaceous species. In French mountains, traditional management of these heathlands involved (and sometimes still involves) prescribed burns at intervals of a few years to a few decades, resulting in a transient re-opening of the environment (Métailié 2006). In this disturbance regime involving alternating levels of competition, a high plasticity of the vegetative apparatus, allowing *A. montana* to maintain itself at the optimum dictated by competition, would be a major advantage. This phenomenon may partly explain why *A. montana* is especially good at colonizing patches created by disturbances such as turf-cutting, a phenomenon described, for example, by Streitberger *et al.* (2022).

The goal of this study was not only to document intraspecific variation in response to management regimes but also to explore how this variability could feed back on the practices of the harvesters. We demonstrated that several traits of particular interest for harvesters are strongly affected by management actions. First, mowing generated a decrease in the vegetative traits that reflect plant biomass, in particular, leaf fresh mass and reproductive height, while we found that reproductive traits were not affected by biomass removal practices such as mowing and grazing. This pattern is expected to largely affect the strategy of the harvesters. Flower-head harvesting depends on reproductive traits only, in particular, on the percentage of rosettes flowering, and should, therefore, not be affected by such management. In contrast, decreased biomass in response to mowing is not favourable for whole-plant harvesters. Personal observations of the authors during harvest show that harvesters do know and take advantage of the trait variability generated by the different management niches, for example, by selecting zones where the plant is taller and ‘thicker’ when harvesting the whole plant. The rate of harvest (in terms of fresh biomass) was reported to be approximately doubled using this strategy. In such zones, harvesters prefer to harvest the whole plant, whereas in other zones where plants are smaller, they limit the harvest to flower heads (J. L. Ardèche 2021, Pyrenees 2022, pers. obs.). This strategy is viable, as the market price for flower heads is approximately three to four times higher than for whole plants, compensating the higher harvesting time required to pick a unit of flower-head biomass compared to picking the whole flowering plant. In contrast to grazing and mowing, tree cover impacted flowering negatively, but was associated with trait values linked to high biomass. Consequently, whole-plant harvesting is expected to be dominant in semi-open environments and clearings in *Fagus sylvatica* and *Pinus sylvestris* stands. This is indeed the case, as many harvesters harvest only the whole plant in such environments, and even specifically target these environments for whole-plant harvesting, as they find the rhizome and roots easier to pull out (J. L. Ardèche 2021, Pyrenees 2022, pers. obs.). Our results not only help us understand how harvesters adapt to intraspecific variation in the phenotype of arnica but they also provide insight on management actions related to this plant. In this context, management actions have so far been analysed mainly according to their impact on the demography of the plant. Here, we argue that, although demographic aspects are undoubtedly of importance, the phenotypic response of arnica to management actions is also of great importance to harvesters. Thus, intraspecific variation should be taken into account when performing management (and in practice it already is). For example, in areas where pastoralism has been abandoned, an intermediate tree cover is expected to be a satisfactory compromise. Tree cover is associated with large arnica plants and reduced dominance of chamaephytes. Dominance by the latter is a main cause of arnica exclusion. Maintaining some tree cover requires less work than complete tree removal. At the same time, reducing tree cover is expected to favour flowering. In line with this prediction, some harvesters in the Pyrenees region have even initiated an operation to cut 30 % of the pine trees present on a site (the tree cutting is distributed throughout the area) in order to increase light availability and arnica flowering, while keeping the other pine trees to maintain the conditions for the shaded phenotype of arnica.

Intraspecific variation in one important functional trait of arnica remains to be studied: the concentration of sesquiterpene lactones, which are the active ingredients in this medicinal plant (Douglas *et al.* 2004). As these compounds function as chemical defences against herbivores and pathogens (Chadwick *et al.* 2013), variation in their concentration is a component of the continuum of plant strategies to tolerate or avoid herbivory (Strauss and Agrawal 1999). Currently, harvesters do not consider variation in concentration of sesquiterpene lactones to be an important variable, as they are paid solely based on the fresh mass harvested. However, this may change as the market evolves. ITV in sesquiterpene lactone content is an important open research question.

To conclude, we demonstrated that management actions strongly affect the phenotype of *A. montana* and that this variability is perceived and used by *A. montana* harvesters to guide their management actions and harvesting practices. By deciphering this feedback loop among management actions, plant traits and harvesting practices, our study may give further insight to harvesters and environment managers about how different types of management actions could be combined in a harvesting site, to create a patchwork of areas managed by mowing and grazing together with unmanaged areas or areas of medium shade, providing opportunities for the different types of harvesting performed by harvesters. Our study calls for a better integration of trait ecology with conservation ecology and stresses the fact that taking into account intraspecific variation, usually not considered in environmental evaluations, could provide useful insights into population monitoring.

## Supporting Information

The following additional information is available in the online version of this article –

Table S1. Summary of model estimates, confidence intervals, likelihood ratio and associated *P* value of the models. Significant effects are given in bold. Soil depth estimates are given per 10-cm unit and elevation per 100-masl unit. CI: 95% confidence interval; LRT, Likelihood ratio test statistic.

Figure S1. Map of the 27 sampling locations. All of them (except the one at lowest altitude) are located in the Parc Naturel Régional des Monts d’Ardèche.

Figure S2. Percentage of the three functional classes (monocotyledons, woody and herbaceous dicotyledons) in the relative cover of each survey plot, in the herb layer only. Colors represent the management practices applied to the site at least once in the past 3 years. Circles surrounded by a thick line indicate quadrats with tree cover > 15 %.

Figure S3. CSR strategies of all arnica individuals sampled. A large proportion (66 %) of the individuals had an S score equal to zero.

Figure S4. First and third components of the PCA of arnica functional traits at the quadrat level. In black: with tree cover; grey: without tree cover and unmanaged (no grazing or mowing); white: without tree cover and managed (grazed or mowed). The centroid of the group is given as a wider point. Height of surrounding vegetation, Grime C, S and R scores and probability of Tephritis presence are shown as supplementary variables.

Figure S5. Correlations between arnica traits of interest (leaf fresh mass, reproductive height, number of flower heads per rosette, percentage of rosettes flowering) and standing biomass.

plad077_suppl_Supplementary_Figures_S1-S5_Table_S1

## Data Availability

The data used to perform the analysis is available at https://doi.org/10.5281/zenodo.10091465.
