# Supplementary material for: A feedback loop between management, intraspecific trait variation and harvesting practices
Source: AoB Plants. 2023 Nov 16;15(6):plad077. doi: 10.1093/aobpla/plad077 (PMC10691405; doi:10.1093/aobpla/plad077)

Supporting information

Table S1: Summary of model estimates, confidence intervals, likelihood ratio and associated p-value of the models. Significant effects are given in bold. Soil depth estimates are given per 10-cm unit, and elevation per 100-masl unit. CI : 95% confidence interval ; LRT : Likelihood ratio test statistic.

Response variable	*Environmental variables*	*Estimate*	*CI*	*LRT*	*p-value*
LA (mm^2)	(Intercept)	14000.00	[8500, 18000]		
	**tree cover**	**750.00**	**[110, 1400]**	**5.20**	**0.05***
	**mowing**	**-1100.00**	**[-1800, -450]**	**9.50**	**0.008****
	**grazing**	**-660.00**	**[-1200, -130]**	**5.80**	**0.05***
	soil depth (/10cm)	-130.00	[-320, 50]	2.10	0.1
	**elevation (/100masl)**	**-650.00**	**[-980, -320]**	**13.00**	**0.002****
SLA ($mm^2.mg^{-1}$)	(Intercept)	32.00	[12, 51]		
	**tree cover**	**7.50**	**[5, 10]**	**23.00**	**<0.001*****
	**mowing**	**-4.20**	**[-6.9, -1.5]**	**8.30**	**0.01***
	**grazing**	**-4.10**	**[-6.2, -2]**	**13.00**	**<0.001****
	**soil depth (/10cm)**	**0.98**	**[0.26, 1.7]**	**6.70**	**0.02***
	elevation (/100masl)	-0.86	[-2.1, 0.43]	1.80	0.2
LDMC ($mg.g^{-1}$)	(Intercept)	150.00	[81, 210]		
	**tree cover**	**-22.00**	**[-30, -13]**	**18.00**	**<0.001*****
	**mowing**	**20.00**	**[11, 30]**	**15.00**	**<0.001*****
	**grazing**	**16.00**	**[9, 23]**	**16.00**	**<0.001*****
	**soil depth (/10cm)**	**-5.10**	**[-7.5,-2.6]**	**13.00**	**<0.001*****
	elevation (/100masl)	1.10	[-3.4, 5.5]	0.24	0.6
LNC (mg.g^{-}1)	(Intercept)	14.00	[-0.84, 30]		
	**tree cover**	**4.00**	**[1.9, 6.1]**	**12.00**	**0.003****
	mowing	-1.50	[-3.6, 0.61]	2.00	0.3
	grazing	-2.10	[-3.8, -0.49]	6.20	**0.04***
	**soil depth (/10cm)**	**0.86**	**[0.29, 1.4]**	**8.00**	**0.02***
	elevation (/100masl)	0.18	[-0.83, 1.2]	0.13	0.7
Leaf fresh mass (mg)	(Intercept)	3800.00	[2300, 5400]		
	tree cover	80.00	[-120, 280]	0.63	0.4

	**mowing**	**-310.00**	**[-530, -90]**	**7.20**	**0.03***
	grazing	-140.00	[-310, 25]	2.80	0.2
	soil depth (/10cm)	-59.00	[-120, -0.17]	3.90	0.1
	**elevation (/100masl)**	**-170.00**	**[-280, -67]**	**9.30**	**0.01***
Leaf dry mass (mg)	(Intercept)	550.00	[340, 760]		
	tree cover	-14.00	[-42, 13]	1.10	0.6
	mowing	-27.00	[-57, 2.2]	3.30	0.2
	grazing	-1.80	[-24, 21]	0.03	0.9
	**soil depth (/10cm)**	**-14.00**	**[-22, -5.8]**	**10.00**	**0.007****
	**elevation (/100masl)**	**-23.00**	**[-37, -9.4]**	**9.60**	**0.008****
Vegetative height (cm)	(Intercept)	35.00	[11, 59]		
	tree cover	1.30	[-1.8, 4.4]	0.71	0.8
	**mowing**	**-5.00**	**[-8.3, -1.6]**	**7.80**	**0.03***
	grazing	-3.10	[-5.7, -0.55]	5.50	0.08.
	soil depth (/10cm)	0.02	[-0.87, 0.92]	0.00	1
	elevation (/100masl)	-1.30	[-2.9, 0.25]	2.80	0.3
Reproductive height (cm)	(Intercept)	120.00	[84, 150]		
	tree cover	4.60	[-0.48, 9.5]	3.20	0.1
	mowing	-5.40	[-10, -0.6]	4.80	0.09.
	grazing	-4.60	[-8.4, -0.84]	5.50	0.07.
	soil depth (/10cm)	-0.20	[-1.5, 1.1]	0.09	0.8
	**elevation (/100masl)**	**-5.30**	**[-7.6, -3]**	**16.00**	**<0.001*****
Flower presence/absence in quadrat (Odds ratio)	**tree cover**	**0.07**	**[0.012, 0.45]**	**8.10**	**0.02***
	mowing	0.97	[0.11, 8.8]	0.00	1
	grazing	1.00	[0.21, 4.8]	0.00	1
	soil depth (/10cm)	1.10	[0.63, 2]	0.17	1
	elevation (/100masl)	0.41	[0.13, 1.4]	2.60	0.4
Mean number of flower heads per rosette	tree cover	×0.53	[0.29, 0.98]	3.90	0.2
	mowing	×0.93	[0.52, 1.7]	0.05	1
	grazing	×0.64	[0.4, 1]	3.50	0.2
	soil depth (/10cm)	×1.10	[0.94, 1.3]	1.50	0.7
	elevation (/100masl)	×0.93	[0.71, 1.2]	0.25	1
Percentage of rosettes flowering	(Intercept)	45.00	[1.8, 1200]		
	**tree cover**	**×0.52**	**[0.33, 0.84]**	**6.60**	**0.05***
	mowing	×0.87	[0.55, 1.4]	0.35	1

	**grazing**	**×0.62**	**[0.44, 0.89]**	**6.20**	**0.05.**
	soil depth (/10cm)	×1.10	[0.95, 1.2]	1.30	0.8
	elevation (/100masl)	×0.93	[0.75, 1.2]	0.38	1
Length of first cauline leaf (cm)	(Intercept)	38.00	[24, 52]		
	tree cover	2.00	[-0.27, 4.4]	3.00	0.08.
	**mowing**	**-3.70**	**[-5.9, -1.6]**	**11.00**	**0.004****
	**grazing**	**-2.90**	**[-4.4, -1.3]**	**11.00**	**0.004****
	**soil depth (/10cm)**	**-0.63**	**[-1.1, -0.11]**	**5.50**	**0.04***
	**elevation (/100masl)**	**-1.80**	**[-2.7, -0.92]**	**12.00**	**0.002****

Figure S1: Map of the 27 sampling locations. All of them (except the one at lowest altitude) are located in the Parc Naturel Régional des Monts d'Ardèche.

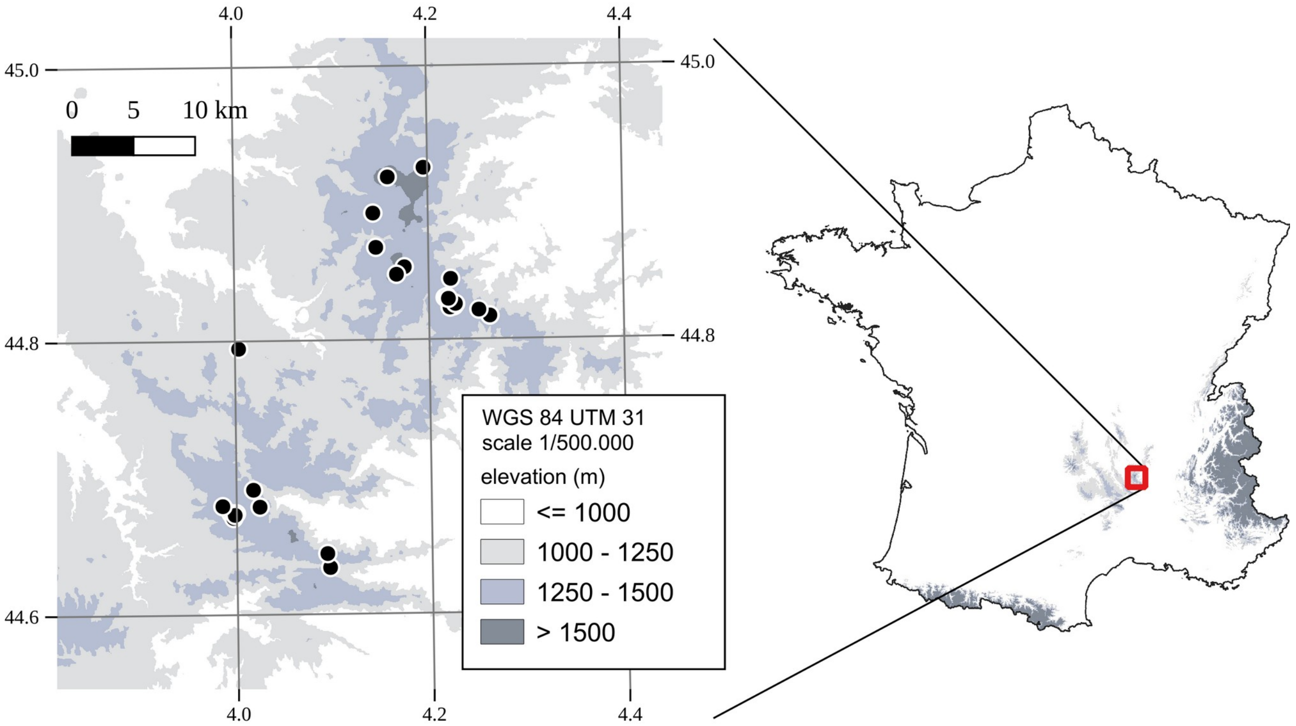

Figure S2: Percentage of the three functional classes (monocotyledons, woody and herbaceous dicotyledons) in the relative cover of each survey plot, in the herb layer only. Colors represent the management practices applied to the site at least once in the past three years. Circles surrounded by a thick line indicate quadrats with tree cover > 15 %.

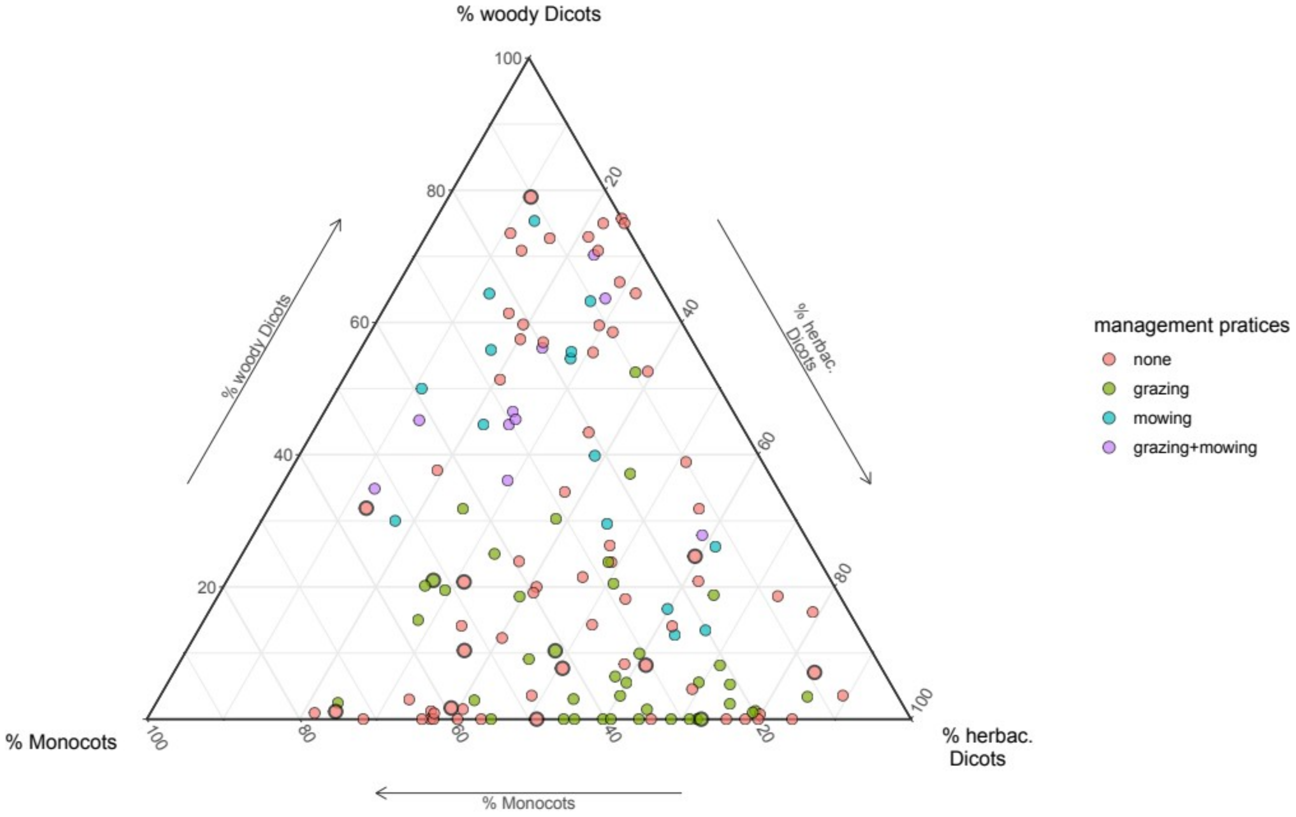

Figure S3: CSR strategies of all arnica individuals sampled. A large proportion (66 %) of the individuals had an S score equal to zero.

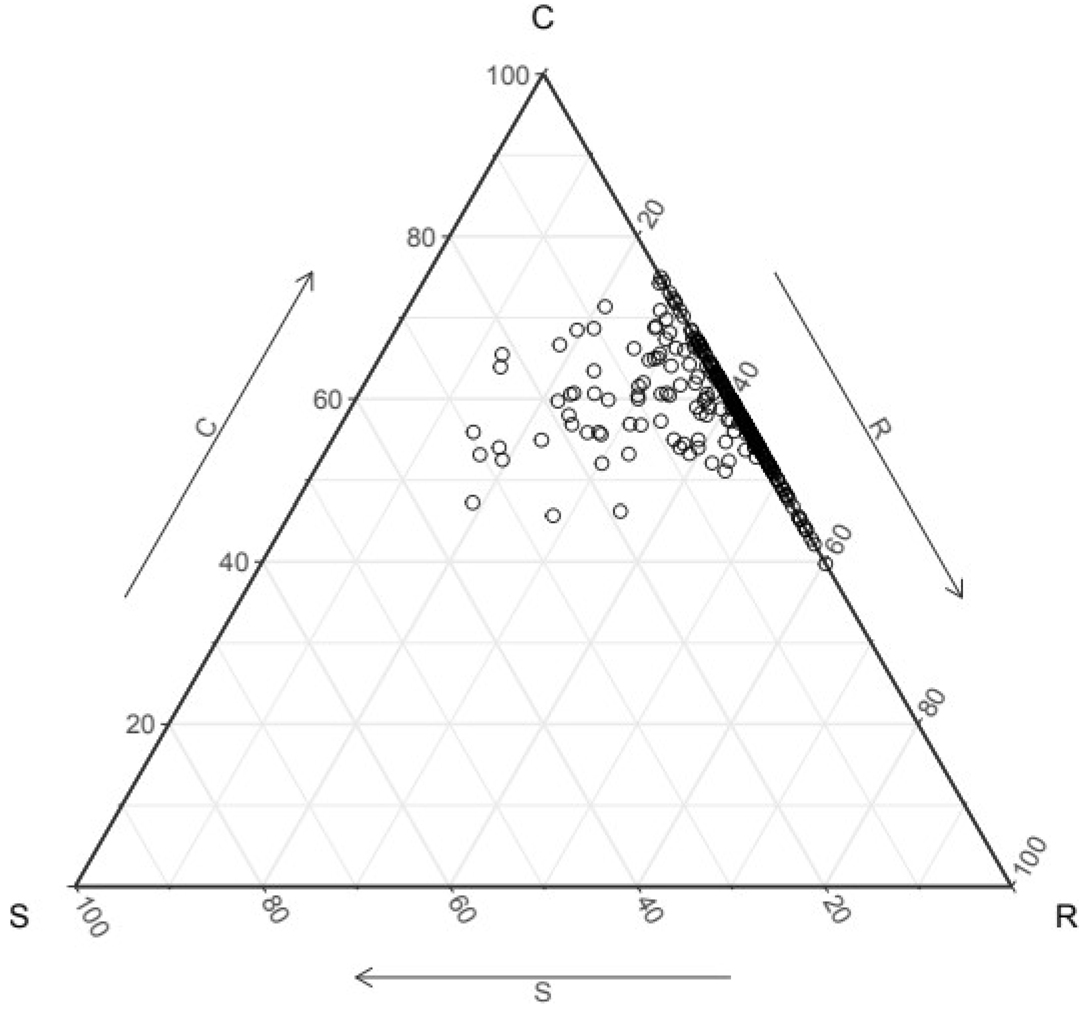

Figure S4: First and third components of the PCA of arnica functional traits at the quadrat level. In black: with tree cover; gray: without tree cover and unmanaged (no grazing or mowing); white: without tree cover and managed (grazed or mowed). The centroid of the group is given as a wider point. Height of surrounding vegetation, Grime C, S and R scores, and probability of *Tephritis* presence are shown as supplementary variables.

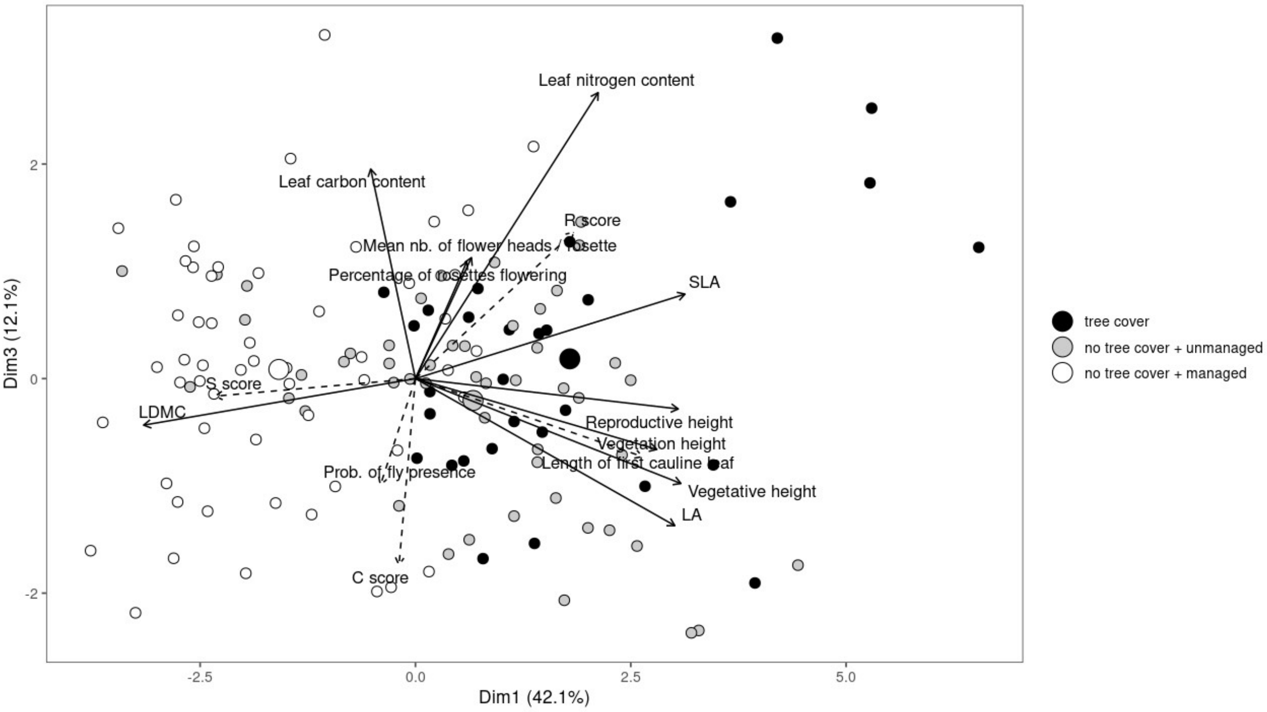

Figure S5: Correlations between arnica traits of interest (leaf fresh mass, reproductive height, number of flower heads per rosette, percentage of rosettes flowering) and standing biomass.

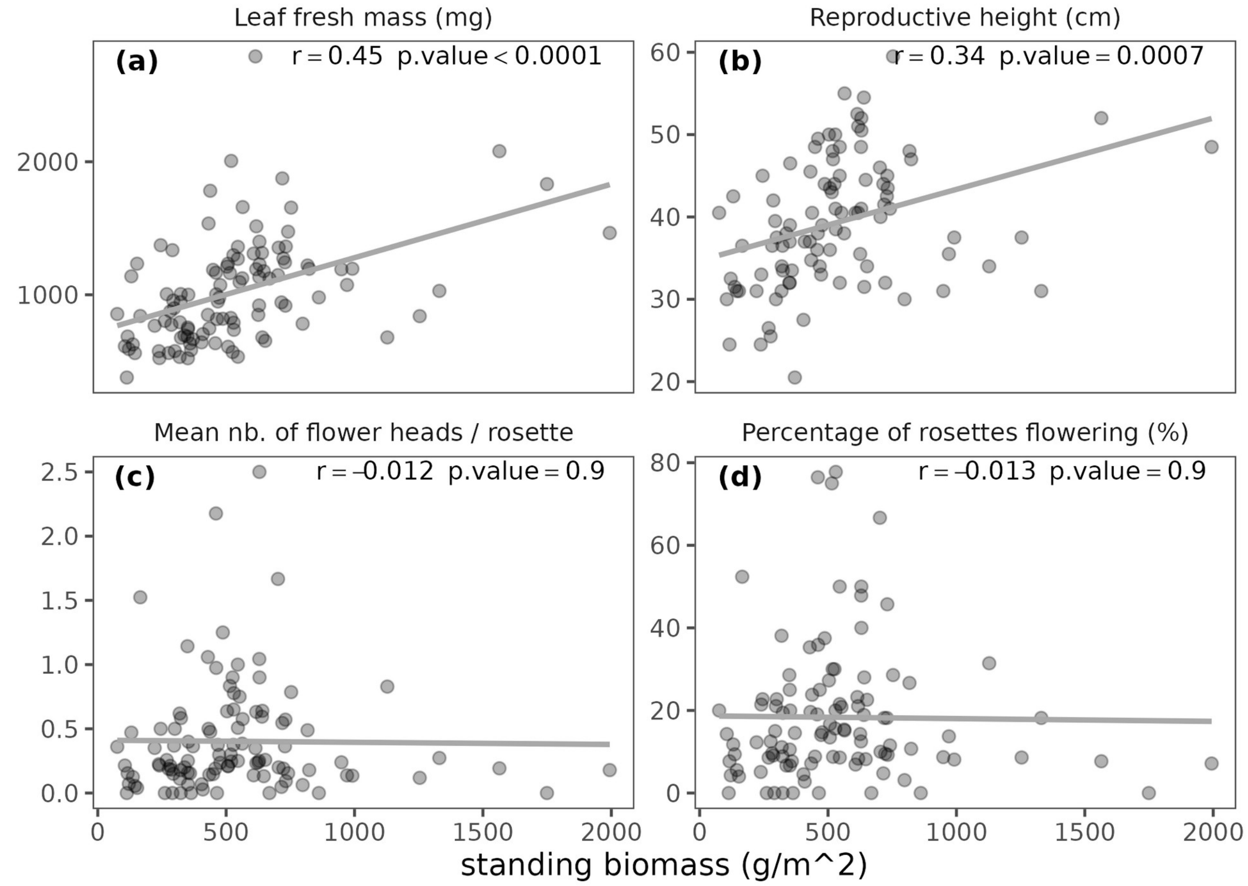

Supplement: plad077_suppl_Supplementary_Figures_S1-S5_Table_S1 [file plad077_suppl_supplementary_figures_s1-s5_table_s1.pdf]
